# Plasticity in the cell division processes of obligate intracellular bacteria

**DOI:** 10.3389/fcimb.2023.1205488

**Published:** 2023-10-09

**Authors:** McKenna Harpring, John V. Cox

**Affiliations:** Department of Microbiology, Immunology, and Biochemistry, University of Tennessee Health Science Center, Memphis, TN, United States

**Keywords:** cell division, obligate intracellular bacteria, peptidoglycan, divisome, elongasome

## Abstract

Most bacteria divide through a highly conserved process called binary fission, in which there is symmetric growth of daughter cells and the synthesis of peptidoglycan at the mid-cell to enable cytokinesis. During this process, the parental cell replicates its chromosomal DNA and segregates replicated chromosomes into the daughter cells. The mechanisms that regulate binary fission have been extensively studied in several model organisms, including *Eschericia coli, Bacillus subtilis*, and *Caulobacter crescentus*. These analyses have revealed that a multi-protein complex called the divisome forms at the mid-cell to enable peptidoglycan synthesis and septation during division. In addition, rod-shaped bacteria form a multi-protein complex called the elongasome that drives sidewall peptidoglycan synthesis necessary for the maintenance of rod shape and the lengthening of the cell prior to division. In adapting to their intracellular niche, the obligate intracellular bacteria discussed here have eliminated one to several of the divisome gene products essential for binary fission in *E. coli*. In addition, genes that encode components of the elongasome, which were mostly lost as rod-shaped bacteria evolved into coccoid organisms, have been retained during the reductive evolutionary process that some coccoid obligate intracellular bacteria have undergone. Although the precise molecular mechanisms that regulate the division of obligate intracellular bacteria remain undefined, the studies summarized here indicate that obligate intracellular bacteria exhibit remarkable plasticity in their cell division processes.

## Mechanisms of cell division in obligate intracellular bacteria

Cell division is a fundamental process for all organisms. In prokaryotes, division is generally accomplished through a highly conserved asexual process called binary fission during which the parental cell replicates its chromosomal DNA and segregates the replicated chromosomes into two daughter cells. The mechanisms that regulate binary fission have been extensively studied in a variety of model organisms including *Eschericia coli, Bacillus subtilis, and Caulobacter crescentus* (Goley et al., 2011; Levin and Janakiraman, 2021; Rohs and Bernhardt, 2021). These studies have revealed that a large multi-protein complex, the divisome, forms at the division plane and orchestrates the steps necessary for septal peptidoglycan synthesis and cell division. Additionally, in rod-shaped bacteria, a multi-protein complex called the elongasome drives sidewall peptidoglycan synthesis necessary for the maintenance of rod shape and the lengthening of the cell prior to septation and cellular constriction (Graham et al., 2021).

Although studies in model organisms have provided a framework for our understanding of the bacterial cell division process and defined the essential gene products that drive it, informatics and mechanistic analyses indicate that obligate intracellular bacteria exhibit remarkable plasticity in their cell division processes. In adapting to their intracellular niche, many obligate intracellular bacteria have eliminated genes associated with various metabolic and stress-induced pathways, as they have become dependent upon a host cell for survival (Sakharkar et al., 2004). However, genes encoding elements of the elongasome apparatus, which were mostly lost as rod-shaped bacteria evolved into coccoid organisms, have been retained during the reductive evolutionary process that some coccoid obligate intracellular bacteria have undergone. One coccoid organism in particular, *Chlamydia trachomatis*, divides by a unique polarized budding process (Abdelrahman et al., 2016) that is dependent upon elements of the elongasome (Ouellette et al., 2012; Abdelrahman et al., 2016). Although the precise molecular mechanisms that regulate the division of obligate intracellular bacteria for the most part remain undefined, genomic studies have revealed the sets of division genes each bacterium has retained. The goal of this review is to provide information on the function of various essential components of the division machinery in the well-studied Gammaproteobacterial model species, *E. coli*, as well as to discuss how gram-negative obligate intracellular bacteria may accomplish division with seemingly incomplete division machinery. For a more comprehensive discussion of cell division in various bacteria see the following manuscripts (Goley et al., 2011; Errington and Wu, 2017; Lutkenhaus and Du, 2017; Du and Lutkenhaus, 2019; Egan et al., 2020; Briggs et al., 2021; Levin and Janakiraman, 2021; Attaibi and den Blaauwen, 2022).

### Peptidoglycan biosynthesis

**Figure 1:**
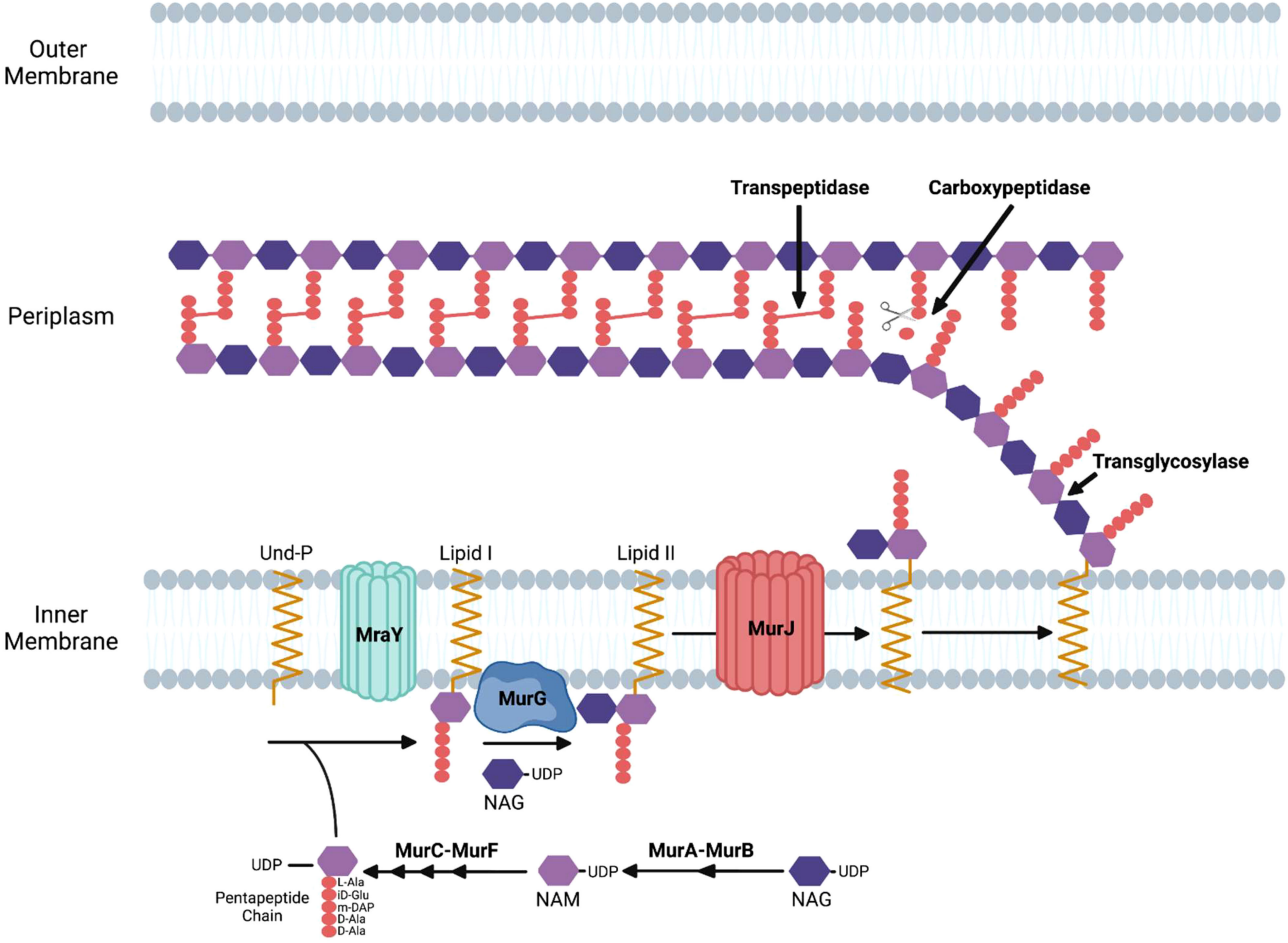
Enzymatic steps of the peptidoglycan biosynthetic pathway in gram-negative bacteria. NAM, N-acetylmuramic acid; NAG, N-acetylglucosamine; Und-P, undecaprenyl phosphate; DAP, diaminopimelic acid.

The process of bacterial cell division in *E. coli* requires the synthesis of peptidoglycan at the septum and in the sidewall through the action of the divisome and the elongasome apparatuses, respectively (Pazos and Peters, 2019). Peptidoglycan is composed of glycan strands with repeating units of the disaccharide *N*-acetylmuramic acid (NAM) and *N*-acetylglucosamine (NAG). The disaccharide building block of peptidoglycan is synthesized in the cytosol where a pentapeptide chain is attached to NAM. This complex is attached to the lipid undecaprenyl phosphate on the cytosolic side of the inner membrane and subsequently flipped to the periplasm where it is added to existing glycan strands via a transglycosylase. Crosslinks between amino acids in the pentapeptide of adjacent glycan strands by transpeptidases stabilize peptidoglycan structure (**Figure 1**) (Kumar et al., 2022).

Peptidoglycan synthesis initiates with fructose-6-phosphate, which is converted to UDP-NAG. The MurA enzyme catalyzes the first committed step of the pathway by transferring enolpyruvate from phosphoenolpyruvate to UDP-NAG. MurB then catalyzes the reduction of enolpyruvate to D-lactate to yield UDP-NAM. The sequential addition of amino acids via the activities of the MurC, MurD, MurE, and MurF results in the formation of a pentapeptide linked to UDP-NAM (**Figure 1**) (El Zoeiby et al., 2003).

The next stages of peptidoglycan biosynthesis occur at the inner membrane in *E. coli*, where the membrane lipid carrier undecaprenyl phosphate carries peptidoglycan precursors through the membrane. Undecaprenyl phosphate in the inner membrane is linked to UDP-NAM with its associated pentapeptide by the MraY enzyme, forming lipid I. MurG is a glycosyltransferase that catalyzes the transfer of NAG to lipid I to produce lipid II (Mohammadi et al., 2007). Although initial studies suggested that FtsW, a member of the SEDS (shape, elongation, division, and sporulation) family of proteins, functions as the transporter that translocates lipid II from the inner to the outer leaflet of the inner membrane in *E. coli* (Mohammadi et al., 2011), more recent data has suggested that MurJ functions as the lipid II flippase in this bacteria (Kuk et al., 2022). Following its transport into the periplasm, transglycosylases transfer the disaccharide precursor to existing glycan strands, and transpeptidases crosslink the pentapeptides in adjacent strands (**Figure 1**).

## The role of the divisome in facilitating septation during binary fission

Most obligate intracellular bacteria divide by binary fission, which is orchestrated by the multi-protein complex called the divisome (Rowlett and Margolin, 2015; Lutkenhaus and Du, 2017). Here we will summarize the role of several divisomal proteins in the binary fission process primarily based on studies in *E. coli*. This will provide a framework for discussing the substantial variation seen in the cell division processes of obligate intracellular bacteria. The components of the divisome accumulate at the septum of dividing cells and are necessary for the segregation of the replicated chromosome into newly formed daughter cells, the synthesis of septal peptidoglycan, and constriction at the mid-cell to enable daughter cell separation. The proteins described below are key elements of the divisome that are essential for the binary fission process in *E. coli*.

### FtsZ

FtsZ is expressed by most bacteria and plays a critical role in orchestrating the steps required for binary fission (Mahone and Goley, 2020; Barrows and Goley, 2021). FtsZ, the prokaryotic homologue of tubulin, assembles into filaments in its GTP-bound state (Mukherjee and Lutkenhaus, 1994). The resulting FtsZ filaments associate laterally to form a Z-ring at the septum of dividing cells. The Z-ring is the first element of the divisome to assemble at the division plane, and it serves as a scaffold for the assembly of other cell division proteins necessary for septal peptidoglycan synthesis and fission.

### Regulating FtsZ positioning

**Figure 2:**
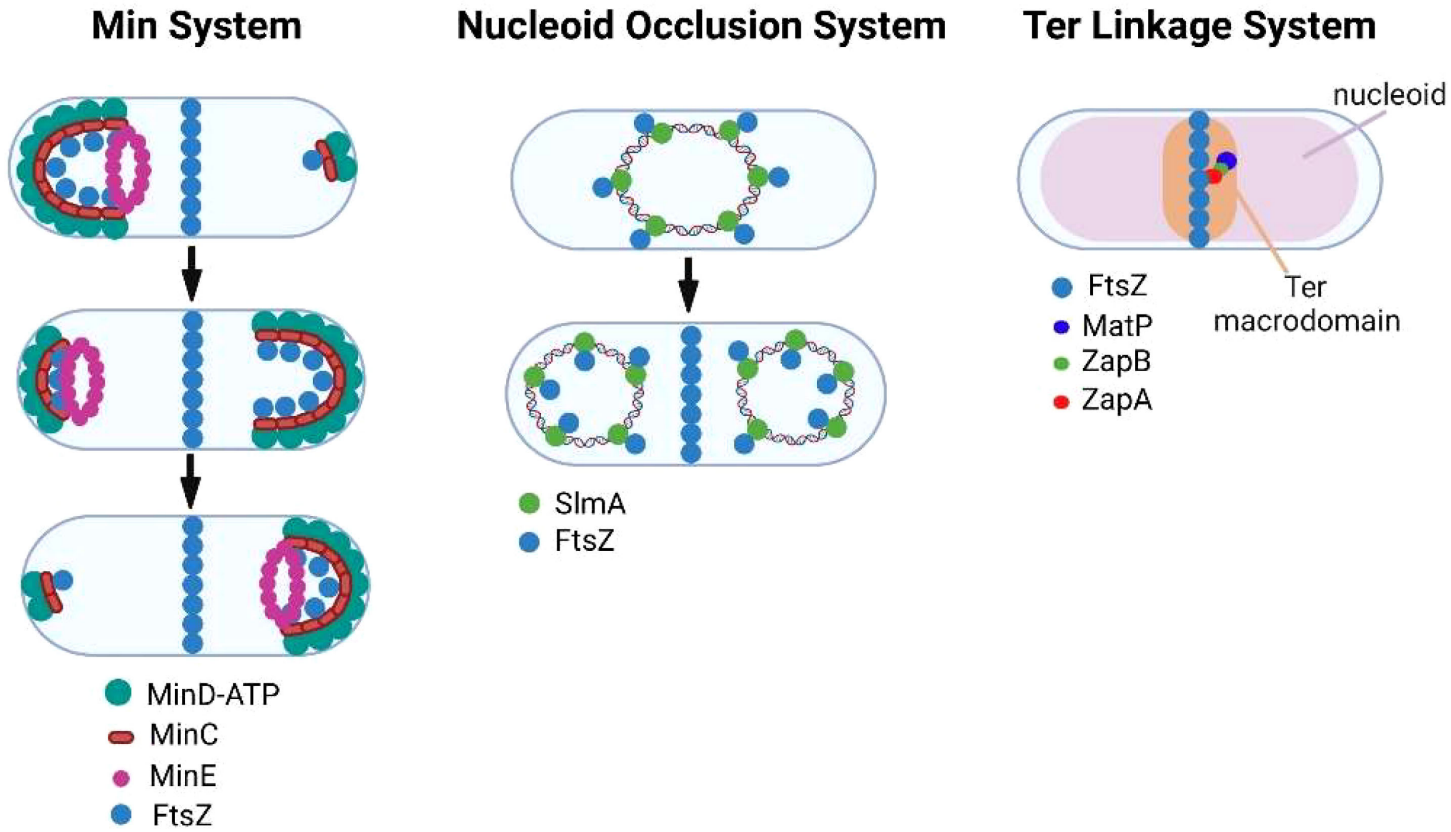
Regulation of FtsZ filament assembly by the Min, nucleoid occlusion, and Ter linkage systems.

Bacteria have evolved multiple mechanisms for ensuring that FtsZ filament assembly is restricted to the division site and its polymerization is inhibited at other locations in the cell. In *E. coli*, the Min system (**Figure 2**) (Wettmann and Kruse, 2018; Ramm et al., 2019) provides spatial regulation of FtsZ assembly through the action of MinC, MinD, and MinE proteins. MinD interacts with the membrane at a pole of the cell where it polymerizes through cooperative binding. MinD then associates with MinC, which functions as a localized FtsZ polymerization inhibitor by binding to FtsZ. The assembly of the MinCD complex is regulated by MinE, which binds MinD and promotes its dissociation from MinC. MinE also stimulates MinD ATPase activity, which results in the release of MinD from the membrane. Following release from the membrane, the MinCD complex assembles at the opposite pole of the cell. The oscillation of the Min system between the poles of the cell inhibits FtsZ filament assembly at the poles and promotes the assembly of FtsZ filaments at the mid-cell (de Boer et al., 1989; Bi and Lutkenhaus, 1993). Several of the obligate intracellular bacteria that are discussed here are members of the Alphaproteobacteria and they do not encode homologues of MinCDE. However, some express a homologue of MipZ a gradient forming member of the ParA/MinD family, which accumulates at the poles of dividing *Caulobacter crescentus* (Kiekebusch et al., 2012). Following its dimerization, MipZ binds to FtsZ monomers and prevents their association with FtsZ filaments (Thanbichler and Shapiro, 2006). In addition, MipZ can cap the plus end of FtsZ filaments promoting their depolymerization (Corrales-Guerrero et al., 2022). These two activities of MipZ suppress FtsZ filament assembly at the poles and result in the preferential accumulation of FtsZ filaments at the mid-cell of dividing *C. crescentus*. Another system employed by rod-shaped bacteria to restrict constriction to the mid-cell is the nucleoid occlusion system (**Figure 2**) (Wu and Errington, 2011; Schumacher, 2017). To prevent the chromosome from being bisected by septum formation during binary fission, the nucleoid occlusion system inhibits septation in regions where the chromosome resides. The protein involved in this inhibitory process in *E. coli* is SlmA, which associates with specific chromosomal sequences and sequesters FtsZ preventing Z-ring formation in the vicinity of its binding sites.

While the Min and nucleoid occlusion systems negatively regulate FtsZ filament assembly, the Ter linkage system is thought to promote FtsZ filament assembly in the vicinity of the *E. coli* replication terminus, which resides near mid-cell prior to FtsZ ring assembly (Bailey et al., 2014). The Ter linkage system is dependent upon MatP, which organizes the Ter macrodomain through its association with specific chromosomal sequences in the vicinity of the replication terminus (Mercier et al., 2008). MatP is proposed to promote FtsZ filament assembly through its interaction with ZapB, which in turn binds the FtsZ-binding protein ZapA thus stabilizing FtsZ filaments in the vicinity of the replication terminus (Galli and Gerdes, 2010; Espeli et al., 2012) (**Figure 2**). A similar Ter linkage system in *C. crescentus* that is dependent upon the DNA binding protein ZapT and the adapter proteins ZauP and ZapA is thought to promote FtsZ filament assembly at the mid-cell of this model alphaproteobacterial organism (Ozaki et al., 2020). Together the Min, nucleoid occlusion, and Ter linkage systems regulate the site of FtsZ polymerization in dividing cells.

### FtsZ treadmilling

FtsZ filaments play a dual role at the septum in recruiting other division proteins to the septum and in generating a contractile force necessary for the initiation of constriction at the mid-cell in some bacterial species (Du and Lutkenhaus, 2019). FtsZ filaments in the Z-ring undergo a treadmilling process in which the assembly of GTP-bound FtsZ occurs at one end of the filament, while filament disassembly promoted by GTP hydrolysis occurs at the other end of the filament (Loose and Mitchison, 2014). FtsZ treadmilling drives its rotational movement at the septum and may be required for the positioning of peptidoglycan biosynthetic enzymes at the division plane (Bisson-Filho et al., 2017; Yang et al., 2017). Although there is no direct evidence for FtsZ treadmilling contributing to constriction at the septum in *E. coli*, studies in *B. subtilis* (Whitley et al., 2021) and *Staphylococcus aureus* (Monteiro et al., 2018) have indicated that early steps in constriction at the septum in these organisms are dependent on this treadmilling process.

## Coordinating chromosomal translocation and septal peptidoglycan synthesis

**Figure 3:**
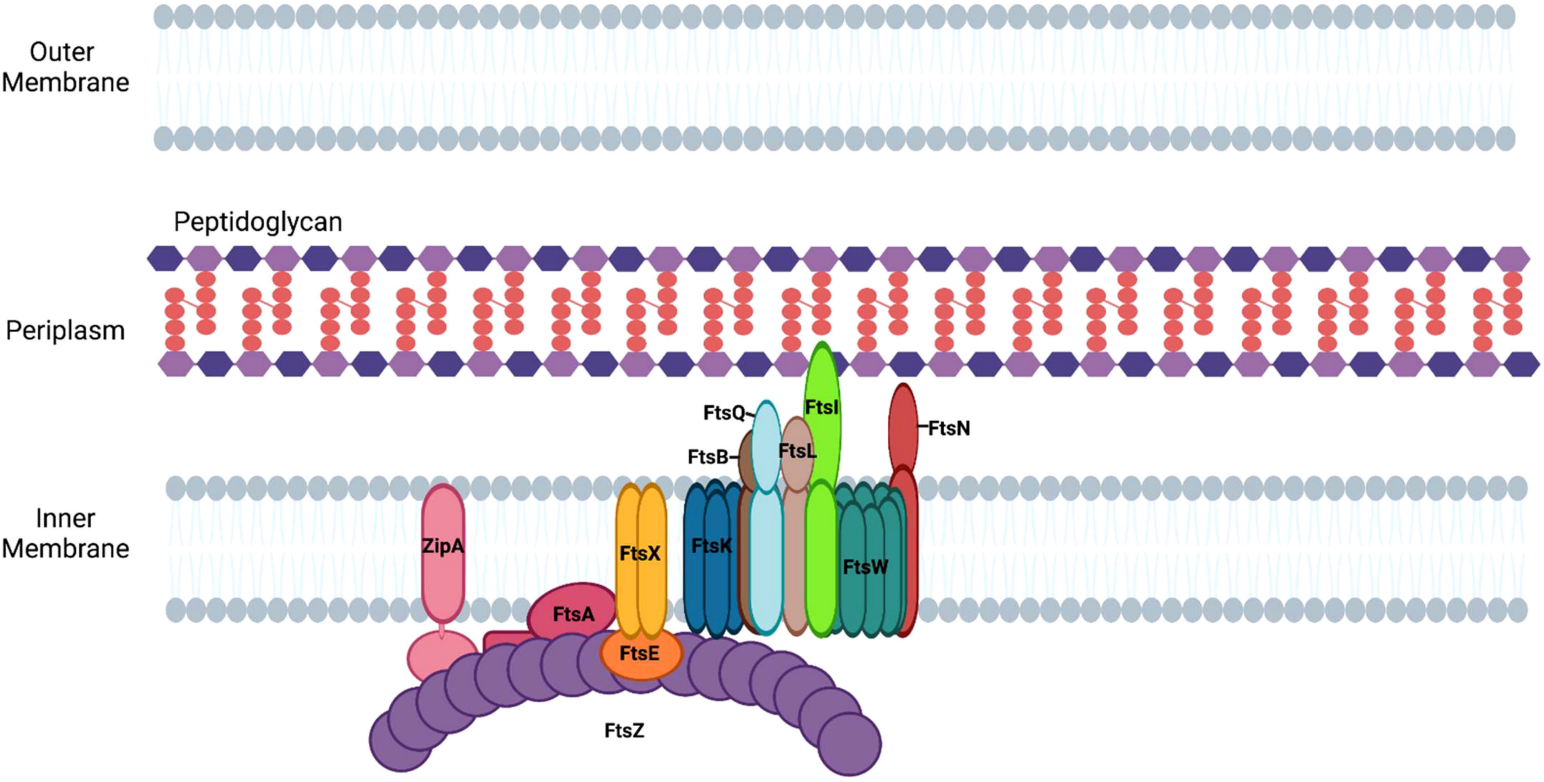
Assembly of divisome proteins at the septum of *E. coli*. Divisome formation in *E. coli* begins with the assembly of FtsZ filaments and ends with the recruitment of FtsN.

Following its polymerization at the mid cell, FtsZ recruits other divisome proteins that each have specialized roles in the division process (Ortiz et al., 2016). The divisome apparatus in *E. coli* is composed of twelve essential proteins, which function together to enable the segregation of the replicated chromosome to each of the newly formed daughter cells, as well as to promote the synthesis of peptidoglycan at the septum (**Figure 3**). The following proteins are considered essential components of the divisome in *E. coli*: FtsZ, FtsA, ZipA, FtsE, FtsX, FtsK, FtsQ, FtsL, FtsB, FtsW, FtsI/PBP3, and FtsN (**Figure 3**). The formation of the divisome apparatus occurs via the sequential recruitment of these proteins, beginning with FtsZ and ending with FtsN (Du and Lutkenhaus, 2017).

### Anchoring FtsZ to the cell membrane

As FtsZ polymers form the Z-ring, the proteins ZipA and FtsA are recruited to the site of division (**Figure 3**) to anchor FtsZ filaments to the cytoplasmic membrane (Barrows and Goley, 2021). FtsA, an actin-like protein, associates with the membrane via an amphipathic helix and binds FtsZ, stabilizing FtsZ filaments at the plane of division (Pichoff and Lutkenhaus, 2005). It does this by anchoring FtsZ to the membrane and promoting its association with ZipA, an integral membrane protein that anchors FtsZ polymers to the membrane at the septum (Hale and de Boer, 1997; Vega and Margolin, 2019). Genetic studies have suggested that FtsA plays an additional role in binary fission by directly activating peptidoglycan synthesis through its interaction with the septal peptidoglycan transglycosylase, FtsW (Park et al., 2021).

### FtsEX

Following the recruitment of FtsA and ZipA to the Z-ring, FtsEX, an ABC transporter family member, is recruited to the divisome through its association with FtsZ (Aarsman et al., 2005; Du et al., 2019). The FtsE subunit of the complex binds to ATP and the activation of the ATPase activity of FtsEX is involved in the activation of septal peptidoglycan synthesis (Pichoff et al., 2019). It has been suggested that FtsX interacts with FtsA and interferes with its polymerization, thereby allowing the monomeric form of FtsA to recruit downstream proteins of the divisome (Du et al., 2016). In addition, ATP hydrolysis by FtsEX positively regulates the activity of amidases, which are hydrolase enzymes that cleave the peptide associated with peptidoglycan (Yang et al., 2011). The periplasmic amidases activated by FtsEX contribute to the separation of daughter cells at the conclusion of division.

### FtsK

Prior to the completion of peptidoglycan synthesis at the septum, the replicated chromosomes are segregated to daughter cells. The divisome protein, FtsK, is recruited to the divisome complex through its interaction with FtsZ and it functions in recruiting other proteins to the divisome and as a DNA translocase (Yu et al., 1998). FtsK not only drives chromosomal translocation during division it also assists in the process of chromosome dimer resolution (Steiner et al., 1999). Following DNA replication, circular chromosomes can become topologically linked by homologous recombination and must be decatenated. The linked chromosome dimers are resolved through the action of the site-specific recombinases, XerC and XerD. The recombinases resolve catenated chromosomes by acting at the *dif* site, which is located near the replication terminus of the chromosome (Blakely et al., 1991). FtsK forms a hexamer and its ATP-dependent motor domain promotes the directional translocation of the chromosome by interacting with KOPS sites (FtsK-Orienting Polar Sequences) in the DNA (Bigot et al., 2005). When FtsK encounters the *dif* site it activates the recombinase activities of XerC and XerD enabling chromosome dimer resolution and the segregation of the replicated chromosomes to the daughter cells (Shimokawa et al., 2013).

### FtsQLB complex

The FtsQLB complex is essential for divisome assembly and is thought to be a direct regulator of PG synthesis (47). FtsQ is initially recruited to the septum in a FtsK-dependent fashion (Chen and Beckwith, 2001) where it interacts with several divisome proteins, including FtsB, FtsW, FtsI, and FtsN (Trip and Scheffers, 2015). FtsB interacts with FtsL and is necessary for formation of the FtsQLB complex (Gonzalez et al., 2010). The cytoplasmic domain of FtsL is required for the recruitment of the peptidoglycan transglycosylase, FtsW, to the divisome (Buddelmeijer et al., 2002; Gonzalez et al., 2010; Marmont and Bernhardt, 2020).

### FtsWI

FtsW, a member of the SEDS (shape, elongation, division, and sporulation) family of proteins, is a monofunctional transglycosylase that catalyzes the addition of disaccharides containing NAG and NAM to existing peptidoglycan strands at the septum. SEDS family members have been shown to be sufficient for both septal and sidewall peptidoglycan synthesis in a subset of bacteria when they are in complex with their cognate transpeptidases (Cho et al., 2016; Meeske et al., 2016; Emami et al., 2017; Reichmann et al., 2019; Taguchi et al., 2019). The recruitment of FtsW to the divisome requires FtsZ, FtsA, FtsQ, and FtsL (Mercer and Weiss, 2002). Once localized at the septum, FtsW recruits FtsI/Pbp3 (Mercer and Weiss, 2002), a transpeptidase that catalyzes 3-4 crosslinks between amino acids in the pentapeptide chains of adjacent glycan strands (Vollmer et al., 2008; Attaibi and den Blaauwen, 2022). In *E. coli*, PBP1b, a nonessential penicillin-binding protein that possesses both transglycosylase and transpeptidase activity, is also recruited to this complex (Attaibi and den Blaauwen, 2022). At this stage of divisome assembly, FtsQLB is hypothesized to inhibit peptidoglycan synthesis by FtsWI until FtsN is recruited to the complex (Attaibi and den Blaauwen, 2022).

### FtsN

FtsI is primarily responsible for the recruitment of the final essential protein of the divisome, FtsN (Wissel and Weiss, 2004). The addition of FtsN results in the initiation of peptidoglycan synthesis and the resulting membrane constriction of the dividing cell. The regulatory role of FtsN in peptidoglycan synthesis appears to be mediated through its interactions with FtsA and the FtsQLB complex (Busiek and Margolin, 2014; Park et al., 2020). Activated FtsA is proposed to directly activate peptidoglycan synthesis by regulating the transglycosylase activity of FtsW (Park et al., 2021), while the interaction of FtsN with the FtsQLB complex is thought to trigger an interaction of the cytoplasmic domain of FtsL with FtsI/PBP3, which stimulates peptidoglycan synthesis by the FtsWI complex (Park et al., 2020). Although FtsN addition to the divisome correlates with the initiation of constriction, the precise mechanism whereby peptidoglycan synthesis drives constriction remains undefined (Gerding et al., 2009; Weiss, 2015).

### Penicillin binding proteins

In addition to the essential components of the divisome, a variety of proteins that contribute to the synthesis, remodeling, and turnover of septal peptidoglycan are expressed in *E. coli*. A subset of these proteins is collectively referred to as penicillin binding proteins (PBPs) as they are targets of ß-lactam antibiotics, and they function as transglycosylases, transpeptidases, carboxypeptidases, and endopeptidases (Sauvage et al., 2008). Transglycosylases catalyze the glycosidic linkages in peptidoglycan, and transpeptidases cross-link amino acids in the pentapeptide chains of adjacent glycan strands. Carboxypeptidases catalyze the removal of the terminal amino acid in the pentapeptide chain, while endopeptidases hydrolyze peptide bonds between non-terminal amino acids in the peptide chain (Sauvage et al., 2008).

The PBPs in *E. coli* are divided into the following classifications according to their size and function. There are three Class A bifunctional PBPs, PBP1a, PBP1b, and PBP1c. Studies in *B. subtilis* initially indicated that these proteins, which possess transglycosylase and transpeptidase activity, are non-essential for growth (McPherson and Popham, 2003). *In vivo* assays have suggested that class A PBPs are not essential for the maintenance of cell shape in *E. coli* (Cho et al., 2016), and PBP1b appears to play a role in maintaining cell wall integrity by repairing cell wall defects (Vigouroux et al., 2020). However, *E. coli* cannot divide in the absence of both PBP1a and PBP1b (Yousif et al., 1985; Denome et al., 1999). The two class B monofunctional PBPs, PBP2 and FtsI/PBP3, possess transpeptidase activity and are essential in *E. coli* for the maintenance of cell shape and cell division, respectively (Spratt, 1975). PBP2 works in concert with its cognate transglycosylase, RodA, to direct sidewall peptidoglycan synthesis (Cho et al., 2016), while PBP3 works in concert with its cognate transglycosylase, FtsW, to direct septal peptidoglycan synthesis (Taguchi et al., 2019). The class C PBPs are the low molecular weight PBPs that possess carboxypeptidase and/or endopeptidase activity and assist in the maturation and recycling of peptidoglycan (Sauvage et al., 2008). The PBPs function in peptidoglycan synthesis or modification at the septum. In addition, these proteins function as components of the elongasome and direct peptidoglycan synthesis or modification associated with cell elongation and maintenance of rod-shape.

### Amidases

During the final stages of division, daughter cells are released from one another through a process that involves constriction of septal peptidoglycan that is linked to the outer membrane. N-acetylmuramoyl-l-alanine amidases function in the periplasm to hydrolyze peptidoglycan cross-links by cleaving the pentapeptide from *N*-acetylmuramic acid (Heidrich et al., 2001; Bernhardt and de Boer, 2003). This facilitates peptidoglycan-dependent constriction at the septum and enables the separation of two newly formed daughter cells.

## The role of the elongasome in facilitating sidewall peptidoglycan synthesis during binary fission

**Figure 4:**
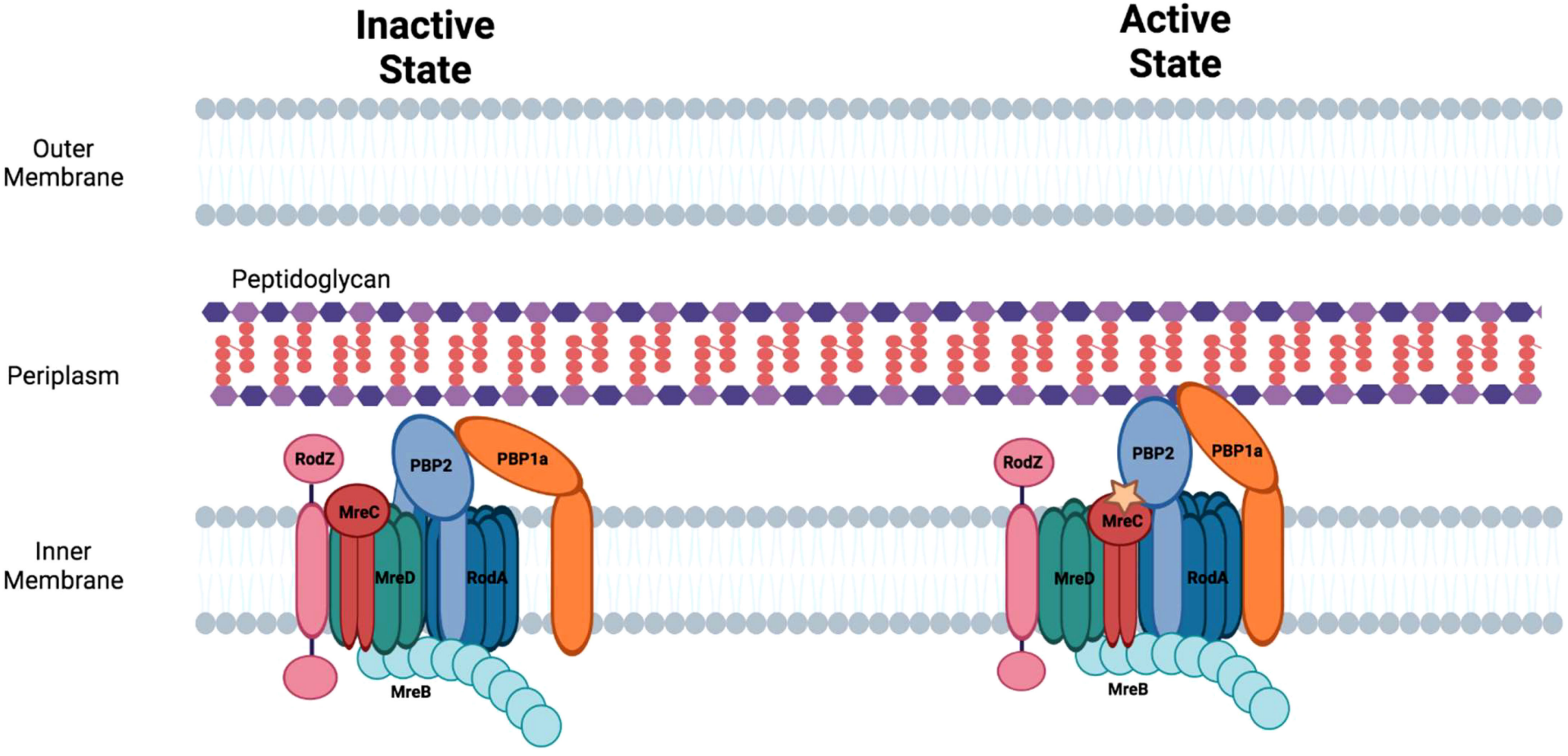
The elongasome apparatus and the potential regulation of sidewall peptidoglycan synthesis. Putative active and inactive peptidoglycan synthetic complexes are shown. Figure was adapted from Liu et al., 2020 (Liu et al., 2020).

In addition to septal peptidoglycan, rod-shaped bacteria synthesize peptidoglycan in their sidewall, which is critical for cell lengthening and the maintenance of shape. Sidewall peptidoglycan synthesis is mediated by the elongasome apparatus (**Figure 4**). The formation of this apparatus is dependent upon components of the Mre system (*mreBCD/rodZ*) which provides a scaffold for the peptidoglycan synthetic complex that contains the transpeptidase, PBP2, and its cognate transglycosylase, RodA (Rohs et al., 2018; Liu et al., 2020). MreB, a prokaryotic homologue of actin, forms filaments on the inner membrane of rod-shaped bacteria (Jones et al., 2001) that are critical for the peptidoglycan synthesis mediated by the elongasome apparatus (Shi et al., 2018). All the gene products that direct the early steps in peptidoglycan synthesis including the flipping of lipid II to the periplasm are shared in common by the divisome and elongasome (Szwedziak and Lowe, 2013). Mutations that inactivate components of the elongasome apparatus result in loss of rod shape and can result in cell death under normal growth conditions (Spratt, 1975; Wachi and Matsuhashi, 1989). However, cells with loss of function mutations in elongasome components can be grown in minimal media at low temperatures (Bendezu et al., 2009). The elongasome proteins and their roles in regulating side-wall peptidoglycan synthesis are discussed below.

### MreB/MreC/MreD

MreB forms multiple, short filaments that move independently around the circumference of rod-shaped bacteria and are involved in directing the sites of peptidoglycan synthesis in the sidewall. The protein contains a conserved amphipathic helix at its N-terminus that allows it to associate with the cell membrane. The MreB-dependent assembly of peptidoglycan in the sidewall enables the cell to maintain cell shape when exposed to osmotic stress. There is a tight coupling between side-wall peptidoglycan synthesis and MreB motility as inhibitors that prevent side-wall peptidoglycan crosslinking also inhibit MreB motility (Dominguez-Escobar et al., 2011; van Teeffelen et al., 2011). A knockout of MreB induces cell rounding and knockout cells eventually lyse likely as a consequence of osmotic stress (Kruse et al., 2005).

MreC forms oligomers (Martins et al., 2021) and associates with multiple components of the elongasome including, MreB, MreD, and PBP2. At least in *Helicobacter pylori*, the association of MreC with PBP2 alters the conformation of this transpeptidase (Contreras-Martel et al., 2017). The change in conformation in PBP2 that is triggered by MreC binding may be necessary for converting PBP2 to its ‘active’ state which directs side-wall peptidoglycan synthesis (**Figure 4**) (Liu et al., 2020).

MreD is another cell shape determining protein that interacts with MreC to regulate peptidoglycan synthesis in the lateral cell wall in rod-shaped bacteria. MreD alters the interaction of MreC with PBP2 potentially maintaining this transpeptidase in an ‘inactive’ state and thereby functioning as a negative regulator of sidewall peptidoglycan synthesis (Liu et al., 2020).

### PBP2/RodA

RodA, another member of the SEDS protein family, is a multi-membrane spanning protein with transglycosylase activity and is essential for the growth of *E. coli* under normal conditions. RodA interacts with the transpeptidase, PBP2, and the activated version of this complex directs side-wall peptidoglycan synthesis (Liu et al., 2020). The circumferential movement of the RodA/PBP2 complex appears to be independent of MreB (Cho et al., 2016). Inactivating mutations in RodA or PBP2 in *E. coli* result in enlarged, spherical cells (Matsuzawa et al., 1973; Spratt, 1975; Meeske et al., 2016). The bifunctional PBP, PBP1a, interacts with PBP2 and is also involved in directing side-wall peptidoglycan synthesis in *E. coli*. PBP1a, which is not essential and appears to function independent of the core elongasome apparatus, may be necessary for filling gaps in peptidoglycan that arise during normal cell growth or following damage (Cho et al., 2016).

### RodZ

RodZ is a transmembrane protein that is recruited to the elongasome through its interaction with MreB (Alyahya et al., 2009), and bacterial two-hybrid studies have shown that it also binds MreC (Bendezu et al., 2009). The interactions of RodZ with MreB and MreC are essential for the maintenance of cell shape. RodZ also interacts with the divisome protein FtsZ (Fenton and Gerdes, 2013; Yoshii et al., 2019) providing a potential link for the transition from cell elongation to cell division, and loss of RodZ function results in a delay in the formation of the divisome apparatus (Yoshii et al., 2019).

### Essential cell division proteins of *C. crescentus*

Several of the obligate intracellular bacteria discussed here are members of the Alphaproteobacteria, and the proteins required for cell division in the model alphaproteobacterial organism, *C. crescentus*, have been extensively analyzed (Singhi and Srivastava, 2020). All the essential divisome gene products of *E. coli* are also essential in *C. crescentus* with the exception of ZipA, which *C. crescentus* does not encode. However, the ordered assembly of the divisome in *C. crescentus* differs from that observed in *E. coli* (Goley et al., 2011). In addition, several essential divisome proteins have been identified in *C. crescentus* that are not expressed in *E. coli* including MipZ, FzlA, and DipI. As described above, MipZ restricts the assembly of the FtsZ ring to the mid-cell in dividing *C. crescentus*, while DipI and FzlA are required for cell constriction during division. DipI is a periplasmic protein that associates with the FtsQLB complex and is essential for the initiation of constriction during division (Osorio et al., 2017). FzlA binds to and controls FtsZ filament curvature and is also essential for constriction at the mid-cell in dividing *C. crescentus* (Lariviere et al., 2018). In the analysis below, we have determined whether the discussed obligate intracellular bacteria possess a homologue of MipZ. FzlA is one of nineteen glutathione S-transferase family members encoded in the *C. crescentus* genome. Although all the obligate intracellular bacteria discussed here contain a single glutathione S-transferase gene, it is unclear whether the protein encoded by this gene functions in a manner similar to FzlA, so it was excluded from the analysis. DipI is composed of two adjacent SH4 domains with an upstream α-helical region. While all the obligate intracellular bacteria discussed here contain a gene that encodes a protein with two SH4 domains, it is again unclear whether this protein is functionally analogous to DipI so it was also excluded from the analysis.

### Cell division in obligate intracellular bacteria

Defining the essential proteins of the divisome and elongasome apparatus in model systems like *E. coli* and *C. crescentus* have provided a framework for understanding the process of growth and division in the gram-negative bacteria discussed here. However, many components of the divisome and elongasome machinery are dispensable for the growth and replication of obligate intracellular bacteria. These organisms have reduced genomes compared to free-living bacteria, as the obligate intracellular bacteria have come to rely on their host cell for their survival (Casadevall, 2008). However, their growth within eukaryotic cells places obligate intracellular bacteria in a position where they are susceptible to the intracellular host innate immune response. Degradation products from the highly conserved peptidoglycan cell wall present pathogen associated molecular patterns (PAMPs) that are recognized by the innate immune receptors, NOD1 and NOD2 (Kanneganti et al., 2007; Rietdijk et al., 2008). Genomic analyses have revealed that some of the obligate intracellular bacteria discussed here lack most or all of the genes required for the synthesis of peptidoglycan precursors (**Table 1**) (Otten et al., 2018; Atwal et al., 2021; Smith et al., 2022), and peptidoglycan was not detected in *Anaplasma phagocytophilum* and *Ehrlichia chaffeensis* using peptidoglycan precursors that could be fluorescently labeled using click chemistry technology (Atwal et al., 2021). In addition, *Chlamydia trachomatis* (Liechti et al., 2014) and *Chlamydia muridarum* (Cox et al., 2020) only transiently synthesize peptidoglycan, which exclusively accumulates at the septum during their cell division processes. Although obligate intracellular bacteria may have reduced peptidoglycan content to more effectively evade detection by the host innate immune response, the peptidoglycan produced by *Rickettsia canadensis*, *Orientia tsutsugamushi, Anaplasma marginale*, and some *Wolbachia pipientis* strains still confers complete or partial resistance to osmotic stress (Atwal et al., 2021).

**Table 1:** Peptidoglycan biosynthetic gene products encoded by the indicated obligate intracellular bacteria.

	*Coxiella*	*Buchnera^A^*	*Buchnera^L^*	*Rickettsia*	*Orientia*	*Wolbachia^AEF^*	*Wolbachia^BCD^*	*Wolbachia^J^*	*Aanaplasma^m^*	*Anaplasma^p^*	*Ehrlichia*	*Chlamydia*
# Genomes	23	38	15	72	8	11	12	2	4	5	15	194
*murA*	100	97	0	100	100	100	100	100	100	0	0	100
*murB*	100	97	0	100	100	100	100	100	100	100	0	100
*murC*	100	97	0	100	100	91	100	100	75	0	0	100
*murD*	100	100	0	99	100	100	100	100	100	0	0	100
*murE*	100	97	53	100	100	100	100	100	100	0	0	100
*murF*	100	29	0	100	100	100	100	100	100	0	0	100
*mraY*	100	97	0	100	100	100	100	100	100	0	0	100
*murG*	100	100	0	100	100	100	100	100	100	0	0	100
*murI*	100	100	0	0	0	0	0	0	0	0	0	0
*murJ*	100	100	40	100	100	100	83	0	100	100	0	100
*alr*	100	0	0	100	0	0	0	0	0	0	0	0
*ddl*	100	71	0	100	100	100	100	100	100	0	0	100

The table indicates the number of genomes that were analyzed and the percentage of sequenced genomes in the Joint Genome Institute database that contained a homologue for each gene. *Buchnera^A^-* endosymbionts of aphids of the subfamily *Aphidinae; Buchnera^L^-*endosymbionts of aphids of the subfamily *Lachinae. Wolbachia^AEF^* (*Wolbachia* supergroups A, E and F)*; Wolbachia^BCD^* (*Wolbachia* supergroups B, C, and D); *Wolbachia^J^* (*Wolbachia* supergroup J)*. Anaplasma^m^-Anaplasma marginale; Anaplasma^p^-Anaplasma phagocytophilum.* **Supplementary File 2**, **Table S2** lists the genomes that were analyzed for each of the obligate intracellular bacteria. Procedures used for genome analysis are described in **Supplementary File 1**.

In addition to variation in their levels of peptidoglycan, none of the obligate intracellular bacteria described here have a complete set of the essential *E. coli* divisome proteins (**Table 2**) (Otten et al., 2018; Atwal et al., 2021; Smith et al., 2022). At present, it is unclear at a molecular level how the divisomal proteins encoded by any of the obligate intracellular bacteria coordinate the steps essential for division. However, it is interesting to note that several of these organisms encode elongasome proteins (**Table 2**) (Otten et al., 2018; Atwal et al., 2021; Smith et al., 2022) even though they are coccoid in morphology (**Table 3**). One of these coccoid organisms, *Chlamydia*, does not encode FtsZ (Stephens et al., 1998) and it has co-opted elements of the elongasome to coordinate its division process (Ouellette et al., 2012; Cox et al., 2020).

**Table 2:** Divisome, elongasome, and peptidoglycan modifying gene products encoded by the indicated obligate intracellular bacteria.

	*Coxiella*	*Buchnera^A^*	*Buchnera^L^*	*Rickettsia*	*Orientia*	*Wolbachia^AEF^*	*Wolbachia^BCD^*	*Wolbachia^J^*	*Aanaplasma^m^*	*Anaplasma^p^*	*Ehrlichia*	*Chlamydia*
# Genomes	23	38	15	72	8	11	12	2	4	5	15	194
*ftsZ*	100	100	100	100	100	100	100	0	100	100	100	0
*ftsA*	100	100	100	100	100	100	100	0	100	100	100	0
*zipA*	100	0	0	0	0	0	0	0	0	0	0	0
*ftsE*	100	0	0	0	0	0	0	0	0	0	0	0
*ftsX*	100	0	0	0	0	0	0	0	0	0	0	0
*ftsK*	100	0	0	100	100	100	100	100	100	100	100	100
*ftsQ*	100	0	0	96	100	100	100	50	100	100	100	99
*ftsL*	100	84	0	100	100	100	0	0	75	0	0	99
*ftsB*	100	95	0	99	100	100	0	0	100	0	0	100
*ftsI*	100	100	0	100	100	100	8	0	100	0	0	100
*ftsW*	100	100	0	100	100	100	8	0	100	0	0	100
*ftsN*	0	0	0	0	0	0	0	0	0	0	0	0
*minC*	0	97	100	0	0	0	0	0	0	0	0	0
*minD*	0	100	100	0	0	0	0	0	0	0	0	0
*minE*	0	100	100	0	0	0	0	0	0	0	0	0
*mipZ*	0	0	0	100	100	0	0	0	0	0	0	0
*slmA*	0	0	0	0	0	0	0	0	0	0	0	0
*mreB*	100	0	0	100	100	100	100	100	100	100	0	100
*mreC*	100	0	0	100	100	0	0	0	100	100	0	100
*mreD*	100	0	0	0	0	0	0	0	0	0	0	0
*pbp2*	100	0	0	100	100	100	100	100	100	0	0	99
*pbp1A,B*	100	66	0	100	0	0	0	0	0	0	0	0
*rodA*	100	0	0	100	100	100	100	100	100	0	0	100
*rodZ*	100	0	0	0	0	0	0	0	0	0	0	100
*amiA,B,C,D*	100	89	0	74	100	0	0	0	0	0	0	99
*dacA,B,C*	100	0	0	100	0	100	100	100	100	100	100	100

The table indicates the number of genomes that were analyzed and the percentage of the sequenced genomes in the Joint Genome Institute database that contained a homologue for each gene. *Buchnera^A^-* endosymbionts of aphids of the subfamily *Aphidinae; Buchnera^L^-*endosymbionts of aphids of the subfamily *Lachinae. Wolbachia^AEF^* (*Wolbachia* supergroups A, E and F)*; Wolbachia^BCD^* (*Wolbachia* supergroups B, C, and D); *Wolbachia^J^* (*Wolbachia* supergroup J)*. Anaplasma^m^-Anaplasma marginale; Anaplasma^p^-Anaplasma phagocytophilum.* **Supplementary File 2**, **Table S2** lists the genomes that were analyzed for each of the obligate intracellular bacteria. Procedures used for genome analysis are described in **Supplementary File 1**.

**Table 3:** Characteristics of the obligate intracellular bacteria.

Genus	Class	Shape	Cellular niche
*Coxiella*	Gammaproteobacteria	rod-shaped	vacuole
*Buchnera*	Gammaproteobacteria	coccoid	vacuole
*Rickettsia*	Alphaproteobacteria	rod-shaped	cytoplasm
*Orientia*	Alphaproteobacteria	coccoid/irregular	cytoplasm
*Wolbachia*	Alphaproteobacteria	coccoid/irregular	vacuole
*Anaplasma*	Alphaproteobacteria	coccoid/irregular	vacuole
*Ehrlichia*	Alphaproteobacteria	coccoid/irregular	vacuole
*Chlamydia*	Chlamydiia	coccoid	vacuole

Informatic studies have demonstrated differences in the array of peptidoglycan biosynthetic and divisome genes retained by gram-negative obligate intracellular organisms from different genera, and between related organisms from the same genus (Otten et al., 2018; Atwal et al., 2021). In addition, variation has been reported in the peptidoglycan biosynthetic and divisome genes retained by different strains of *Buchnera aphidicola* and *Wolbachia pipientis* (Otten et al., 2018; Atwal et al., 2021; Smith et al., 2022). Our analysis, which is summarized in **Tables 1**, **2**, has confirmed these observations and documented additional variability in the essential *E. coli* divisome gene products retained by different strains of *B. aphidicola* and *W. pipientis*. How organisms with minimal peptidoglycan biosynthetic and/or divisome machinery potentially accomplish cell division is discussed below.

### Cell division in *Coxiella* spp.

*Coxiella* are gram-negative rod-shaped bacteria (**Table 3**) that like *E. coli* are members of the Gammaproteobacteria. C. *burnettii*, the most prevalent *Coxiella* species is transmitted through aerosols and preferentially infects mononuclear phagocytes. When the bacteria spreads to humans, it can lead to Q-fever, a disease that can present as flu-like or can result in a chronic condition that can lead to endocarditis (Dragan and Voth, 2020). C. *burnettii* can also infect cells of the placenta in pregnant women and induce premature deliveries or stillbirths (Carcopino et al., 2009).

*Coxiella* are internalized by host cells through a microfilament-dependent parasite-directed endocytic pathway and they reside within a parasitophorous vacuole in the host (Heinzen et al., 1999; Minnick and Raghavan, 2012; Dragan and Voth, 2020). Although *Coxiella* divide by binary fission, they lack the genes encoding components of the Min and nucleoid exclusion systems (**Table 2**). Like all the obligate intracellular bacteria discussed here, *Coxiella* also have an incomplete Ter linkage system, and the mechanisms that direct FtsZ filament assembly at the mid-cell during division in *Coxiella* are unclear. These organisms undergo a biphasic developmental cycle that generates distinct large cell variants that undergo replication and small cell variants that are the non-replicating stationary phase form of the organism. Both large and small cell variants can infect cells *in vitro*, although the small cell variant is likely the initiator of natural infections *in vivo* and it is able to survive harsh environmental conditions (Dragan and Voth, 2020).

*Coxiella* encode a homologue for all the essential proteins of the *E. coli* divisome apparatus except for FtsN (**Table 2**), which plays a key regulatory role in switching on peptidoglycan synthesis in *E. coli* (Weiss, 2015). An FtsN homologue is not encoded by any of the obligate intracellular bacteria described here indicating that these organisms have developed alternative mechanisms to switch on septal peptidoglycan synthesis during division. *Coxiella* possess a peptidoglycan sacculus and they possess all the *E. coli* genes necessary to synthesize peptidoglycan (**Table 1**). In addition to the PBP1, PBP2, and PBP3 transpeptidases (**Table 2**), *Coxiella* express L,D-transpeptidases that introduce non-classical 3-3 peptide crosslinks into peptidoglycan that are critical for the association of peptidoglycan with β-barrel outer membrane proteins (Sandoz et al., 2021). Interestingly, the expression of these L,D-transpeptidases is elevated in small cell variants (Sandoz et al., 2016) and crosslinks introduced by these proteins into peptidoglycan may be critical for cell wall stability and enable *Coxiella* to survive harsh environmental conditions (Minnick and Raghavan, 2012).

### Cell division in *Buchnera* spp.

*Buchnera*, like *E. coli*, are Gammaproteobacteria within the order Enterobacterales. There is a single species in the genus, *Buchnera aphidicola.* These gram-negative bacteria are obligate endosymbionts in aphid insects. Unlike the other obligate intracellular bacteria discussed in this review, the insect host of these bacteria cannot survive in their absence (Shigenobu and Wilson, 2011). Members of *Buchnera* are coccoid and divide by binary fission within a vacuole (**Table 3**) in a specialized cell in the infected host called a bacteriocyte (Shigenobu and Wilson, 2011). As *Buchnera* strains have co-evolved with their aphid hosts, they have retained homologues of very different subsets of the essential *E. coli* divisome genes. As an example of this, most *Buchnera* that are endosymbionts of aphids of the subfamily *Aphidinae* have retained *ftsZ*, *ftsA*, *ftsB*, *ftsL, ftsW*, and *ftsI* (*Buchnera^A^* in **Table 2**), while *Buchnera* that are endosymbionts of aphids of the subfamily *Lachinae* (*Buchnera^L^* in **Table 2**) have only retained *ftsZ* and *ftsA. Buchnera* are the only obligate intracellular bacteria discussed here that encode homologues of MinCDE (**Table 2**), which likely position the FtsZ ring at the mid-cell during the binary fission process. The *Buchnera^A^* and *Buchnera^L^* strains are also the only obligate intracellular bacteria discussed here that do not encode FtsK, and the mechanisms that direct chromosome translocation during binary fission are unclear.

Early studies indicated that *Buchnera* have a classical peptidoglycan sacculus (Houk et al., 1977) and are sensitive to β-lactam antibiotics (Griffiths and Beck, 1974). However, the *Buchnera^A^* and *Buchnera^L^* strains differ in the essential *E. coli* peptidoglycan biosynthetic genes that they encode (**Table 1**). While some of the *Buchnera^A^* strains encode the majority of the peptidoglycan biosynthetic genes, a subset of *Buchnera^A^* strains do not encode MurC, MurE, and MurF, and none of the *Buchnera* strains analyzed here encode the alanine racemase (Alr) (**Table 1**). Recent studies using peptidoglycan precursors that could be fluorescently labeled using click chemistry have demonstrated that one of the *Buchnera^A^* strains that does not encode MurC, MurE, and MurF can synthesize peptidoglycan (Smith et al., 2022). The ability of this strain to synthesize peptidoglycan was not dependent upon the presence of co-symbionts in the infected aphid, and it was suggested that the remaining Mur gene products encoded by this strain may have acquired ligase activities to compensate for the loss of MurC, MurE, and MurF (Smith et al., 2022). While the *Buchnera^L^* strains have retained FtsZ and FtsA, they lack all the transglycosylases and transpeptidases that direct peptidoglycan synthesis and crosslinking in the periplasm (**Table 2**). The majority of the *Buchnera^L^* strains have also lost all of the genes necessary for the synthesis of peptidoglycan precursors. How these organisms complete the binary fission process in the absence of peptidoglycan and with a minimal divisome apparatus is not understood.

### Cell division in *Rickettsia* spp. and *Orientia* spp.

*Rickettsia*, and *Orientia* are Alphaproteobacteria within the family *Rickettsiaceae*. These organisms have retained a similar array of the *E. coli* gene products that direct cell division and peptidoglycan biosynthesis and they will be discussed together. *Rickettsia* spp. are gram-negative bacilli (**Table 3**) that cause a range of diseases including, Rocky Mountain spotted fever, rickettsial pox, epidemic typhus, and murine typhus (Matos et al., 2022). *Rickettsia* are transmitted by an arthropod host that feeds on mammals and the transmitted bacteria target the microvascular endothelium, leading to endothelial dysfunction. Host cell receptors interact with *Rickettsia* ligands resulting in adhesion of the organism, which undergoes phagocytosis. In *Rickettsia parkeri* (Borgo et al., 2022) and *Rickettsia typhi* (Rahman et al., 2013), Pat1 phospholipase A_2_ promotes the release of the internalized organism from the phagosome into the cytosol of the infected cell where it replicates. Since all sequenced *Rickettsia* species contain a gene encoding Pat1 (Rahman et al., 2013; Borgo et al., 2022), this enzyme may enable phagosomal membrane release for all members of the *Rickettsia*.

*Orientia* is a genus within the *Rickettsiaceae* family. These organisms are coccoid/irregular-shaped (**Table 3**), and there are two known human pathogenic members of the genus, O. *tsutsugamushi* and O. *chuto*. *Orientia* are transmitted to humans through larval stage mites, and this can result in the development of scrub typhus (Salje, 2017). Following entry into a host cell by endocytosis, *Orientia* escape the endosome and move to a perinuclear location where they replicate by binary fission until ~fifty bacterial cells reside within the host. The replicated bacterial cells then exit the host by a budding process that results in the bacterium being released in a vesicle derived from the host cell plasma membrane (Banerjee and Kulkarni, 2021).

The *Rickettsia* and *Orientia* divide by binary fission and they possess homologues of the same subset of the essential *E. coli* divisome genes including *ftsZ*, *ftsA*, *ftsK*, *ftsQ, ftsL, ftsB, ftsW, and ftsI* (**Table 2** – only genomes from O. *tsutsugamushi* were included in our analysis). These organisms also encode a homologue of MipZ, which likely positions the FtsZ ring at the mid-cell during cell division (**Table 2**). In addition to the divisome gene products, these bacteria express homologues of all the *E. coli* genes required for peptidoglycan biosynthesis with the exception of *murI*, a glutamate racemase that converts L-glutamate to D-glutamate (**Table 1**). MurI is not expressed by any of the obligate intracellular bacteria discussed here that are members of the Alphaproteobacteria. At least in the *Rickettsia*, however, an alternative pathway gives rise to D-glutamate as this amino acid has been detected in peptidoglycan isolated from these organisms (Pang and Winkler, 1994). In addition to lacking MurI, the *Orientia* do not encode the alanine racemase, Alr (**Table 1**). Peptidoglycan has not been biochemically characterized in the *Orientia*, and these organisms are insensitive to β-lactam antibiotics (Fenollar et al., 2003). However, microscopic analyses have demonstrated the presence of peptidoglycan in *Orientia* metabolically labeled with fluorescent peptidoglycan precursors (Atwal et al., 2017; Atwal et al., 2021). The basis for the insensitivity of these bacteria to β-lactam antibiotics is unclear at this time. While the *Rickettsia* and *Orientia* encode homologues of many of the components of sidewall peptidoglycan synthesis machinery including, MreB, MreC, RodA, and PBP2 (**Table 2**), the *Rickettsia* are rod-shaped and the *Orientia* are coccoid/irregular in shape (**Table 3**). Recent analyses have postulated that this difference in morphology could be due to the expression of class A PBPs in the *Rickettsia* (**Table 2**), which could affect the abundance and/or crosslinking of peptidoglycan and ultimately the morphology of the cells (Atwal et al., 2021).

### Cell division in *Wolbachia* spp.

*Wolbachia* are Alphaproteobacteria in the family *Anaplasmataceae.* These bacteria are transmitted through the germline, but also can colonize some somatic tissues of insects and arthropods. Inflammatory filarial disease in humans results from the passage of worm larvae by mosquitoes during a blood meal (Dietrich et al., 2019). *Wolbachia* are coccoid/irregular shaped organisms and they grow and divide by binary fission within a host cell vacuole (**Table 3**). These organisms are capable of surviving for a short time outside of a host cell. However, they cannot synthesize essential lipids and therefore are unable to divide (Pietri et al., 2016).

Strains of the single species of *Wolbachia, Wolbachia pipientis*, are distributed in several clades referred to as supergroups. Fourteen supergroups (A-O) have been described that infect arthropods and nematodes (Liu et al., 2023). These supergroups are not species but reflect different evolutionary lineages that arose from a primary separation between the two most abundant clades, Supergroups A and B. For the purposes of this review, we have grouped supergroups A, E, and F (*Wolbachia^AEF^*) and B, C, and D (*Wolbachia^BCD^*) together based on the similar array of divisome genes the supergroup members have retained. Members of the *Wolbachia^AEF^* supergroups encode homologues of the divisome gene products FtsZ, FtsA, FtsK, FtsQ, FtsL, FtsB, FtsI, FtsW and the elongasome proteins MreB, PBP2, and RodA (**Table 2**). In contrast, most members of the *Wolbachia^BCD^* supergroups do not encode a homologue of FtsL, FtsB, the FtsI transpeptidase, or the FtsW transglycosylase. The *Wolbachia^BCD^* organisms have retained homologues of the elongasome components RodA and PBP2 (**Table 2**) suggesting that these components may direct septal peptidoglycan synthesis and crosslinking in the absence of FtsW and FtsI. *Wolbachia^J^* supergroup members, which have undergone an even more dramatic reduction in the composition of their divisome, do not encode a homologue of FtsZ. Although *Wolbachia^J^* supergroup members have lost almost all the essential genes of the *E. coli* divisome, they still encode MreB, RodA, and PBP2 (**Table 2**), again suggesting a role for these elongasome components in directing septal peptidoglycan synthesis during binary fission. The absence of FtsZ in the *Wolbachia^J^* supergroup members further suggests the possibility that MreB may substitute for FtsZ to coordinate septal peptidoglycan synthesis and crosslinking in these bacteria. While *Wolbachia^J^* supergroup members encode most of the genes required for the synthesis of lipid II peptidoglycan precursors (**Figure 1**), they do not encode MurJ or FtsW, the two enzymes that have been linked to the transport of lipid II precursors from the cytosol to the periplasm for incorporation into growing glycan stands at the septum of dividing cells (Mohammadi et al., 2011; Kuk et al., 2022). Whether the SEDS protein family member, RodA (Egan et al., 2020; Kumar et al., 2022), potentially functions in translocating lipid II into the periplasm of *Wolbachia^J^* supergroup members is unclear at this time.

### Cell division in *Anaplasma* spp.

*Anaplasma* are Alphaproteobacteria in the family *Anaplasmataceae*. These bacteria are tick-transmitted pathogens that can infect myeloid cells, erythrocytes, leukocytes, or endothelial cells in cattle and cause the disease granulocytic anaplasmosis. *Anaplasma* are coccoid/irregular shaped, gram-negative bacteria that grow and divide within a parasitophorous vacuole in the infected host cell (Severo et al., 2012) (**Table 3**). The organism has two developmental forms. The dense cored morphotype binds to and is internalized by host cells. Following internalization, the dense cored morphotype differentiates into a reticulate cell, which divides by binary fission in the parasitophorous vacuole (Blouin and Kocan, 1998).

Individual species of the genus *Anaplasma* have undergone significant divergence with respect to their divisome and peptidoglycan biosynthetic machinery. *Anaplasma marginale* (*Anaplasma^m^* in **Tables 1**, **2**) encodes an array of divisome and peptidoglycan biosynthetic gene products very similar to those observed in the *Rickettsia*, *Orientia*, and supergroups A, E and F of the *Wolbachia*. In contrast, *Anaplasma phagocytophilum* (*Anaplasma^p^* in **Tables 1**, **2**) has only retained the genes for the divisome components FtsZ, FtsA, FtsK, and FtsQ, and the peptidoglycan biosynthetic gene products MurB and MurJ. Peptidoglycan was not detected in *Anaplasma phagocytophilum* using peptidoglycan precursors that could be fluorescently labeled using click chemistry (Atwal et al., 2021). Except for MreB and MreC, *Anaplasma phagocytophilum* also does not encode any of the components of the elongasome. In the absence of peptidoglycan, *Anaplasma phagocytophilum* incorporates cholesterol from the host cell into their membranes, which is thought to provide structural rigidity to the cell and it is required for the organism to infect target cells (Lin and Rikihisa, 2003). While it has been suggested that *Anaplasma phagocytophilum* divides by binary fission, microscopic analyses have demonstrated the presence of irregular buds on the surface of this organism (Lin and Rikihisa, 2003). Although it is unclear whether these buds represent intermediates in cell division, it is interesting to note that FtsZ-less *E. coli* that are referred to as L-forms can divide in media with reduced osmolarity, and these cells appear to give rise to daughter cells by a process of irregular budding from their cell surface (Mercier et al., 2016).

### Cell division in *Ehrlichia* spp.

*Ehrlichia* are Alphaproteobacteria in the family *Anaplasmataceae*. These organisms are coccoid/irregular shaped, gram-negative bacteria that invade monocytes, granulocytes, lymphocytes, or platelets within vertebrates and grow within cytoplasmic vacuoles (**Table 3**). *Ehrlichia* spp. take on two forms during the process of infecting a vertebrate host cell: small dense-cored cells (DCs) with a condensed nucleoid and larger reticulate cells (RCs) with a more uniformly dispersed nucleoid. DCs attach to a host cell and are internalized by endocytosis (Zhang et al., 2007). Following their uptake, DCs are converted to RCs, which replicate within a vacuole in the host. The replicating organisms form large aggregates within the vacuole called *morulae* (Dedonder et al., 2012).

*Ehrlichia* spp. have retained a small subset of the essential divisome proteins of *E. coli*. These organisms encode homologues of FtsZ, FtsA, FtsK, and FtsQ, and they do not encode any of the components of the elongasome (**Table 2**). In addition, *Ehrlichia* spp. do not contain any of the essential gene products required for the synthesis of peptidoglycan precursors in *E. coli* (**Table 1**), and peptidoglycan was not detected in *E. chafeensis* using peptidoglycan precursors that could be fluorescently labeled using click chemistry (Atwal et al., 2021). Like *A. phagocytophilum*, *E. chaffeensis* incorporate cholesterol from the host into their cell envelopes, and this membrane cholesterol is necessary for the organism to infect target cells (Lin and Rikihisa, 2003). While it has been suggested that *Ehrlichia* divide by binary fission, microscopic analyses have demonstrated the presence of irregular buds on the surface of *E. chaffeensis* (Lin and Rikihisa, 2003) that may correspond to intermediates in the cell division process.

### Polarized cell division in *Chlamydia* spp.

*Chlamydiae* are a diverse group of gram-negative bacteria that infect a broad spectrum of species (Elwell et al., 2016; Collingro et al., 2020). This review will primarily focus on the cell division processes of the human pathogen, *Chlamydia trachomatis.* Infection of epithelial cells of the genital tract by this organism is the leading bacterial cause of sexually transmitted disease and can lead to infertility (Darville and Hiltke, 2010; O’Connell and Ferone, 2016). In addition, these organisms infect cells of the eye and are the leading cause of preventable blindness worldwide (Taylor et al., 2014). Like all members of the *Chlamydiae, Chlamydia trachomatis* undergo a biphasic developmental cycle during the course of infection. A non-dividing elementary body (EB) infects a host cell and differentiates into a replicative reticulate body (RB) within a parasitophorous vacuole called an inclusion (**Table 3**). Following RB multiplication within the inclusion, RBs re-differentiate into EBs, which are released from the cell to initiate another round of infection.

**Figure 5:**
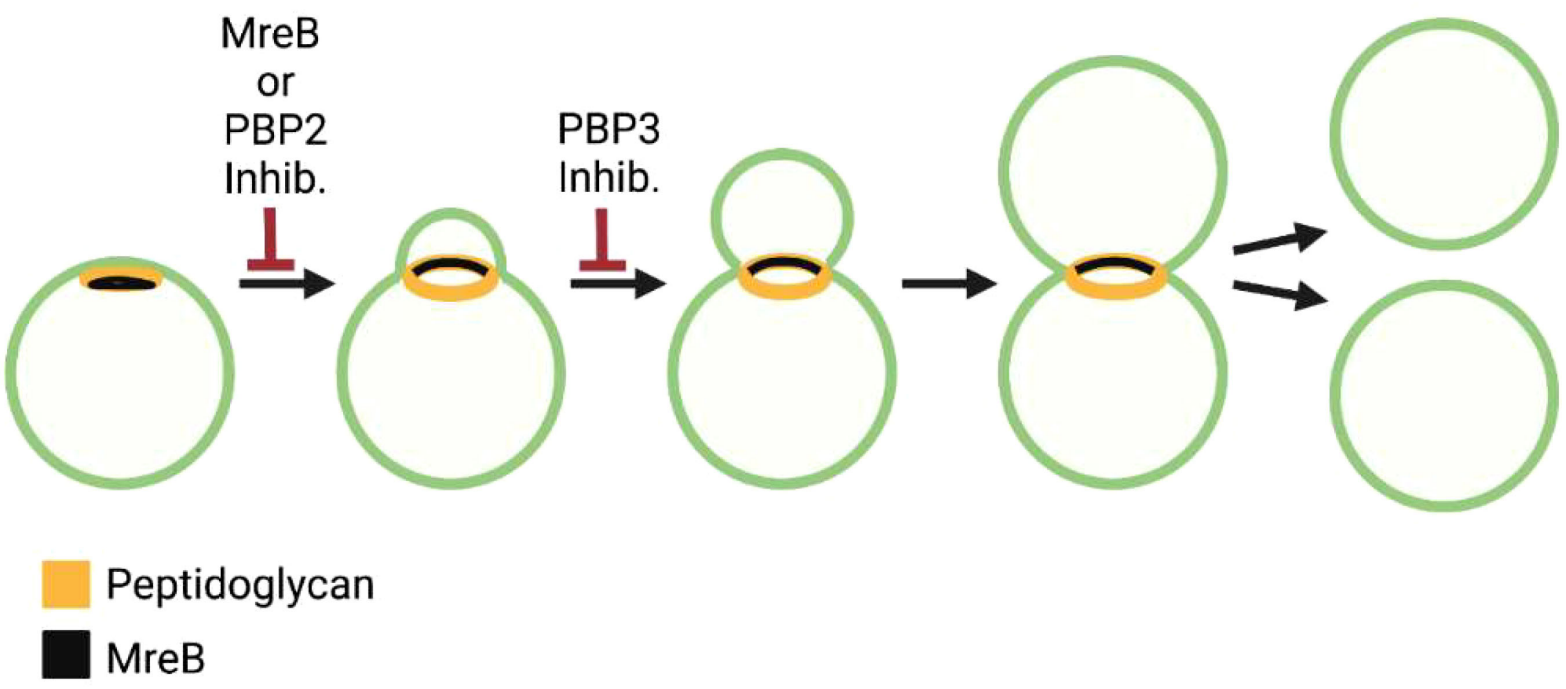
Steps in the polarized cell division of the *C. trachomatis.* Distribution of peptidoglycan and MreB and the effect of MreB, PBP2, and PBP3 inhibitors on the chlamydial division process are shown.

Genome sequence analysis has revealed that *Chlamydia trachomatis* does not encode a homologue for FtsZ (Stephens et al., 1998), the essential regulator of cell division in most bacteria. Although some *Chlamydiae* are thought to divide by binary fission (Bayramova et al., 2018), imaging analyses have indicated that *Chlamydia trachomatis* and *Chlamydia muridarum*, members of the *Chlamydiaceae*, divide by a novel polarized budding process (Abdelrahman et al., 2016; Cox et al., 2020). This budding process is characterized by an asymmetric expansion of the membrane from one pole of a coccoid cell that results in the formation of a nascent daughter cell (**Figure 5**). *Chlamydiae* are members of the Chlamydia/Verrucomicrobia/Planctomycetes superphyla. Planctomycetes are free living organisms that also lack FtsZ. Similar to Chlamydia, two different modes of cell division have been observed for members of the Planctomycetota phyla. Bacteria from the class Planctomycetia divide by budding, while those from Phycisphaerae divide by binary fission (Wiegand et al., 2020).

Peptidoglycan has been isolated and characterized from *Protochlamydia amoebophilia* (Jacquier et al., 2015), a member of the order *Chlamydiales*. However, researchers have been unable to isolate peptidoglycan from members of the family *Chlamydiaceae*, even though these organisms are sensitive to ß-lactam antibiotics and possess the majority of genes required for peptidoglycan biosynthesis (**Table 1**). This paradox, which was referred to as the ‘chlamydial anomaly’ (Moulder, 1991) was resolved when studies using peptidoglycan synthesis precursors that could be fluorescently labeled demonstrated the presence of peptidoglycan that exclusively accumulated at the septum of dividing *Chlamydia trachomatis* (Liechti et al., 2014), and *Chlamydia muridarum* (Cox et al., 2020). This finding was corroborated by mass spectrometry studies that detected intermediates in peptidoglycan synthesis and/or degradation in *Chlamydia trachomatis* serovar L2 (Packiam et al., 2015).

*Chlamydia* are coccoid organisms and they encode homologues of the *E. coli* divisome components FtsK, FtsQ, FtsL, FtsB, FtsW, and FtsI/PBP3 (**Table 2**), and localization studies have shown that chlamydial FtsQ accumulates at the septum in cells undergoing polarized budding (Abdelrahman et al., 2016). In addition to these divisome components, *Chlamydia* encodes homologues of many of the elongasome gene products including MreB, MreC, RodZ, PBP2, and RodA (**Table 2**). Studies using MreB inhibitors have indicated that the initiation of chlamydial cell division is dependent upon MreB (**Figure 5**) (Ouellette et al., 2012; Abdelrahman et al., 2016), and it was hypothesized that MreB may coordinate divisome assembly in these FtsZ-less organisms (Ouellette et al., 2012; Ouellette et al., 2020). It was subsequently demonstrated that MreB and RodZ accumulate at the septum of dividing *Chlamydia* (Kemege et al., 2015; Liechti et al., 2016; Lee et al., 2020), and MreB function was required for the assembly of septal peptidoglycan rings (Liechti et al., 2016). A role for MreB in coordinating septal divisome assembly in *Chlamydia* was further supported by the interesting observation that the co-expression of chlamydial MreB and RodZ at least partially complements an FtsZ deficiency in *E. coli* (Ranjit et al., 2020).

Studies using peptidoglycan precursors that can be fluorescently labeled revealed that one of the early events in the division of *Chlamydia trachomatis* and *Chlamydia muridarum* is the formation of a patch of peptidoglycan at one pole of the cell (**Figure 5**) (Cox et al., 2020). As the daughter cell emerges from this pole of the cell, the peptidoglycan patch is converted to a peptidoglycan ring (**Figure 5**). Inhibitor studies indicated that peptidoglycan regulates at least two steps in this polarized division process. Cells treated with inhibitors that prevent peptidoglycan synthesis or peptidoglycan crosslinking by PBP2 are unable to initiate polarized division, while cells treated with inhibitors that prevent peptidoglycan crosslinking by FtsI/PBP3 initiate polarized division, but the process arrests at an early stage of daughter cell growth (**Figure 5**) (Cox et al., 2020). These results suggest that components of the elongasome machinery (MreB and PBP2) act upstream of the divisome component, PBP3, in regulating the polarized division process of *Chlamydia*.

MreB exhibits a polar distribution in coccoid *Chlamydia* prior to the onset of division and at the septum of cells undergoing division (**Figure 5**) (Liechti et al., 2016; Lee et al., 2020). Recent studies have demonstrated a critical role for cardiolipin in directing the localization of MreB in the chlamydial division process. Cardiolipin is synthesized in a polar fashion in dividing *Chlamydia*, and an inhibitor that disrupts cardiolipin-rich membrane microdomains alters the restricted localization of MreB in dividing cells (Ouellette et al., 2022). It will be interesting in future studies to determine whether MreB localization in *Chlamydia* is regulated via a direct interaction with cardiolipin or is dependent upon the negative membrane curvature associated with cardiolipin-rich membrane microdomains (Renner and Weibel, 2011).

There is currently no additional information regarding the role of FtsK, FtsL, FtsW, RodA, or MreC in regulating chlamydial cell division, but the recent development of genetic tools, especially conditional knockdowns using CRISPRi technology (Ouellette et al., 2021), hold great promise for further dissecting the role of these proteins in regulating the polarized budding of these organisms. It is interesting to note that studies in *Planctopirus limnophila*, a budding ovoid member of the FtsZ-less Planctomycetes, revealed that knockouts of FtsW, FtsI, and MreB did not affect the growth rate of these free-living organisms (Rivas-Marin et al., 2020). However, investigators were unable to establish a knockout of the chromosomal translocase, FtsK, suggesting it is essential for growth (Rivas-Marin et al., 2020). FtsK has been retained as part of the cell division machinery of all the obligate intracellular bacteria discussed above with the exception of the *Buchnera* (**Table 2**). Whether FtsK is essential for the assembly of the divisome and the segregation of replicated chromosomes during chlamydial cell division will be addressed in future studies.

## Conclusion

**Figure 6:**
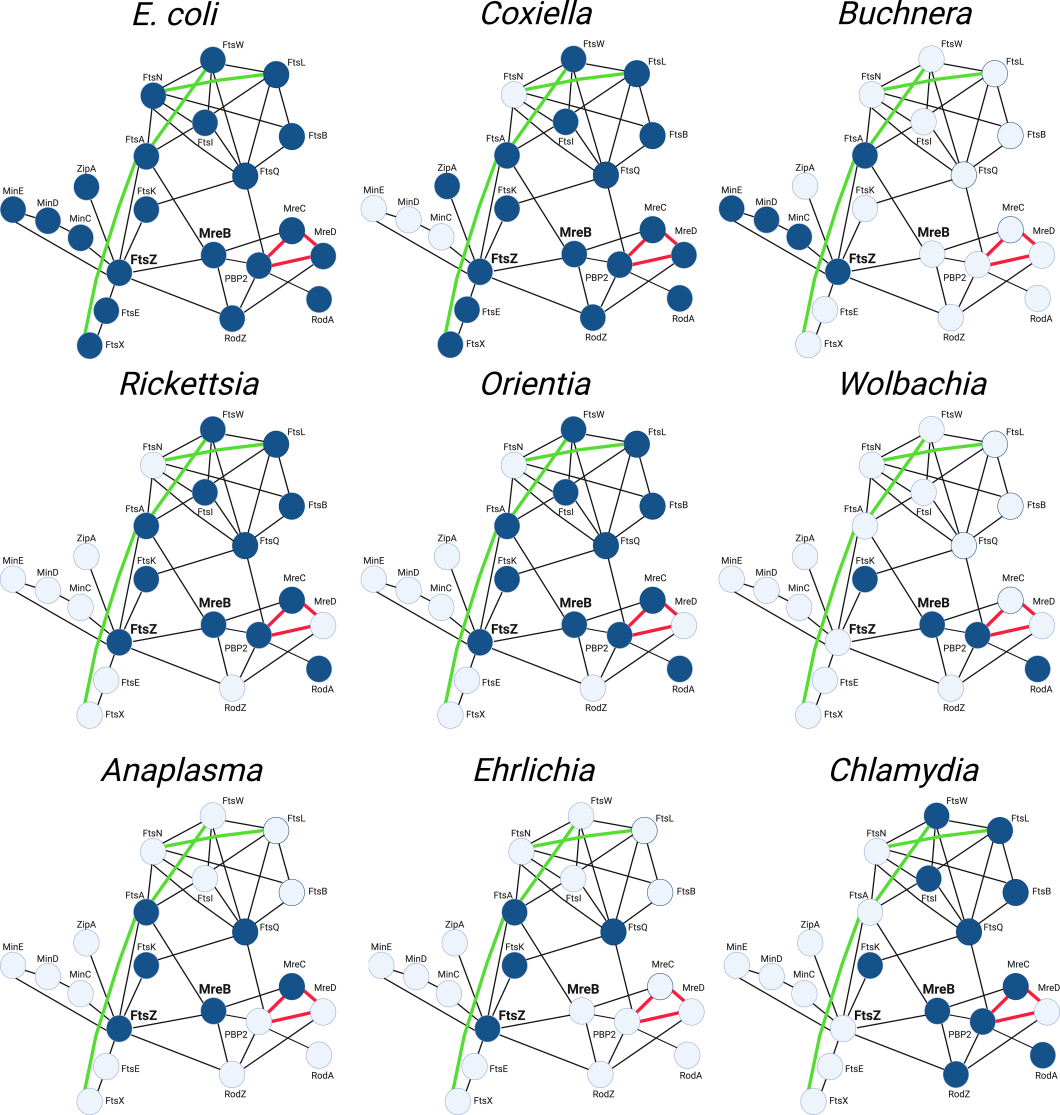
String maps (Szklarczyk et al., 2021) illustrating protein-protein interactions of the divisome and elongasome machinery of the indicated bacteria. The interaction maps of the various obligate intracellular bacteria indicate the gene products they have retained (highlighted blue) and their putative interactions based on studies from *E. coli*. The maps shown for the *Buchnera*, *Wolbachia*, and *Anaplasma* illustrate the genes retained by the *Buchnera^A^* strains of *Buchnera aphidicola*, a *Wolbachia* pipientis strain from supergroup J, and *Anaplasma phagocytophilum*, respectively (see **Table 2**). The interactions indicated with black lines are based on two-hybrid studies in *E. coli*. The green lines represent recently identified genetic interactions in *E. coli* (Du et al., 2016; Park et al., 2020; Park et al., 2021). The red lines represent interactions characterized in *E. coli* using FRET technology (Liu et al., 2020).

The mechanisms that regulate the cell division process of *E. coli* have been extensively investigated. These studies have led to the protein interaction map (Szklarczyk et al., 2021) depicted in **Figure 6** that illustrates known protein-protein interactions in *E. coli* and the potential interplay between elements of the divisome and elongasome during the growth and division of this organism. **Figure 6** also depicts the array of gene products retained (highlighted in blue) by some of the obligate intracellular bacteria discussed here and their potential interactions as they have adapted to their intracellular lifestyle. Although the molecular mechanisms that regulate the division of these obligate intracellular bacteria are for the most part undefined, these putative interaction maps suggest that a wide array of FtsZ-dependent division processes occur in obligate intracellular bacteria. Some of these organisms, such as *Coxiella* spp., are likely to undergo division in a manner similar to the processes characterized in *E. coli*. However, organisms such as the *Ehrlichia* and some of the *Buchnera*, *Wolbachia* and *Anaplasma* (**Figure 6**), have eliminated most of the division machinery of *E. coli* and in some cases the ability to synthesize peptidoglycan (**Tables 1**, **2**) yet they have retained the ability to divide.

The interaction maps in **Figure 6** further illustrate that the components of the divisome and elongasome interact in *E. coli*, and these interactions may coordinate the processes of cell growth and division (Ago and Shiomi, 2019; Yoshii et al., 2019). The *Chlamydiae* do not encode a homologue for FtsZ, and they have co-opted elements of the elongasome to direct their cell division process (Ouellette et al., 2012; Cox et al., 2020). The MreB-dependent division of these organisms, at least among members of the *Chlamydiaceae*, is characterized by a novel polarized budding process, and recent studies have suggested that interactions between membrane phospholipids and the MreB-dependent cytoskeleton of *C. trachomatis* may be critical for regulating their polarized membrane growth (Ouellette et al., 2022). Our informatic analysis revealed that members of the *Wolbachia^J^* supergroup also do not encode FtsZ, but they have retained the elongasome components MreB, RodA, and PBP2. These organisms may represent another example where elements of the elongasome substitute for divisome proteins to direct septal peptidoglycan synthesis and crosslinking during cell division. Although members of the *Chlamydiae* and *Wolbachia^J^* supergroup members are the only obligate intracellular bacteria that do not encode FtsZ, all the bacteria discussed in this review have lost genes essential for cell division in *E. coli* as they have adapted to their intracellular lifestyle. Most of these organisms do not encode FtsN and FtsEX, critical regulators of peptidoglycan synthesis and remodeling in *E. coli*, and how these organisms regulate peptidoglycan synthesis and breakdown during their division processes are not defined. Informatic analyses further indicate that some of the obligate intracellular organisms lack the ability to synthesize peptidoglycan (**Table 1**). The mechanisms that regulate division in organisms that lack the capacity to synthesize peptidoglycan are unclear. However, A. *phagocytophilum* and *E. chafeenis*, which do not produce detectable peptidoglycan (Atwal et al., 2021), exhibit irregular buds on their surface that may represent intermediates in cell division (Lin and Rikihisa, 2003). While many questions remain unanswered, the continued development of molecular tools holds great promise for dissecting how obligate intracellular bacteria with major differences in their cell division machinery coordinate the processes of membrane growth, chromosome segregation, septum formation, and fission that are necessary to divide. The information obtained from these studies may eventually lead to the development of alternative therapeutic approaches for inhibiting the growth of these bacteria, many of which are human pathogens.

## Author contributions

MH wrote the article JC wrote and edited the article. All authors contributed to the article and approved the submitted version.
