# Supplementary material for: Plasticity in the cell division processes of obligate intracellular bacteria
Source: Front Cell Infect Microbiol. 2023 Oct 9;13:1205488. doi: 10.3389/fcimb.2023.1205488 (PMC10591338; doi:10.3389/fcimb.2023.1205488)
Supplement: Supplementary file 1 [file DataSheet_1.pdf]

The bacterial species that were characterized in this review are listed in Supplemental File 2, Table S1. The genome sequences of these bacteria (Supplemental File 2, Table S2) were acquired from Integrated Microbial Genomes and Microbiomes (IMG/M) [1] and include genomes sequenced by the Joint Genome Institute at DOE as well as those from GenBank and the Sequence Read Archive. These sequences were queried for genes encoding proteins homologous to components of the divisome and elongasome, and for genes encoding proteins homologous to the enzymes that direct peptidoglycan biosynthesis and modification. Only finished genomes with a M2 Completeness score [2] of greater than 98% were used for the analysis. Genomes were analyzed using the IMG Genome Blast algorithm with a cut off E-value of 1e-5. The bacterial genomes were searched for homologues of the genes from *Escherichia coli* K-12 MG1655 and *Caulobacter vibrioides* NA1000 listed in Supplemental File 2, Table S3. The gene loci that encode homologues of the divisome, elongasome, and peptidoglycan biosynthetic gene products in the various obligate intracellular bacteria are listed in Supplemental File 2, Table S4. If homologues were identified in some but not all species or strains of a genus, the genomes were reanalyzed using the sequence from a reference genus member (bolded in Supplemental File 2, Table S2) using a cut off E-value of 1e-30. Homologues containing less than 60% of the protein sequence as compared to the other homologues in the genus were not scored as a functional gene product. Genes that met the above criteria of a functional gene but were not annotated in the IMG/M analysis software were further queried using Keggs Taxonomy Mapping and Protein Tables from National Center for Biotechnology Information (NCBI) Genome Resource. The genes encoding chlamydial FtsB, FtsL, and FtsQ have not been annotated in either of these resources, but these gene products have been characterized in publications [3, 4].

The taxonomy of the hosts for the *Buchnera*^{A} and *Buchnera*^{L} strains included in this analysis are listed in Supplemental File 2, Table S5. The taxonomy of the hosts for the *Wolbachia*^{AEF}, *Wolbachia*^{BCD}, and *Wolbachia*^{J} members included in the analysis are listed in Supplemental File 2, Table S6. See Vancaester et al. 2023 [5] for a description of the *Wolbachia* supergroups.

References:

[1] Chen IA, Chu K, Palaniappan K, Ratner A, Huang J, Huntemann M, et al. The IMG/M data management and analysis system v.7: content updates and new features. Nucleic Acids Res. 2023;51:D723-D32.
[2] Chklovski A, Parks DH, Woodcroft BJ, Tyson GW. CheckM2: a rapid, scalable and accurate tool for assessing microbial genome quality using machine learning. Nat Methods. 2023;20:1203-12.
[3] Kaur H, Lynn AM. Mapping the FtsQBL divisome components in bacterial NTD pathogens as potential drug targets. Front Genet. 2022;13:1010870.
[4] Ouellette SP, Rueden KJ, AbdelRahman YM, Cox JV, Belland RJ. Identification and Partial Characterization of Potential FtsL and FtsQ Homologs of Chlamydia. Front Microbiol. 2015;6:1264.
[5] Vancaester E, Blaxter M. Phylogenomic analysis of Wolbachia genomes from the Darwin Tree of Life biodiversity genomics project. PLoS Biol. 2023;21:e3001972.

I-Min A Chen and others, The IMG/M data management and analysis system v.7: content updates and new features, *Nucleic Acids Research*, Volume 51, Issue D1, 6 January 2023, Pages D723–D732, https://doi.org/10.1093/nar/gkac976

Vancaester E, Blaxter M. Phylogenomic analysis of Wolbachia genomes from the Darwin Tree of Life biodiversity genomics project. PLoS Biol. 2023 Jan 23;21(1):e3001972. doi: 10.1371/journal.pbio.3001972. PMID: 36689552; PMCID: PMC9894559.
